# Encapsulation and Degradation Kinetics of Bioactive Compounds from Sweet Potato Peel During Storage

**DOI:** 10.17113/ftb.58.03.20.6557

**Published:** 2020-09

**Authors:** Vanja Šeregelj, Gordana Ćetković, Jasna Čanadanović-Brunet, Vesna Tumbas Šaponjac, Jelena Vulić, Slađana Stajčić

**Affiliations:** University of Novi Sad, Faculty of Technology, Bulevar cara Lazara 1, 21 000 Novi Sad, Serbia

**Keywords:** sweet potato peel, bioactve compounds, encapsulation, storage stability, kinetic degradation

## Abstract

**Research background:**

The aim of this work is to evaluate utilization of sweet potato peel as a source of bioactive compounds. The effect of solvents (acetone and acetone/ethanol mixture) on the extraction efficiency of total carotenoids and phenolics from sweet potato tuber, flesh and peel, and antioxidant activity were investigated. Sweet potato peel extract stood out in terms of antioxidant activity and was chosen for encapsulation by spray and freeze-drying.

**Experimental approach:**

Encapsulation is an effective method to improve phytochemical stability by entrapping the core material with a coating agent. In this study, spray and freeze-drying techniques were applied for improving the stability of bioactive compounds (carotenoids and phenolics) using whey protein as a coating material. The main advantages of the applied techniques over the other encapsulation techniques are simplicity, continuity, effectiveness, availability and applicability.

**Results and conclusions:**

Physicochemical characteristics revealed that spray drying resulted in the formation of lower size particles, better flow properties and encapsulation efficiency of carotenoids. The retention of encapsulated and non-encapsulated bioactive compounds was monitored during storage in daylight and dark conditions. Storage conditions affected the carotenoid retention, whereas higher degradation rate of all samples was observable in daylight. Phenolic compounds exhibited higher retention in all investigated samples. Degradation kinetic parameters suggest the longer shelf life of spray dried encapsulated extract and potent method for stabilization of bioactive ingredients.

**Novelty and scientific contribution:**

This study demonstrates that the spray drying technique and utilization of sweet potato peel have a big potential for the development of functional additives with improved nutritional, colour and bioactive properties.

## INTRODUCTION

Sweet potato (*Ipomoea batatas* L.) is increasingly recognized as a health food that contains various phytochemicals which help to reduce the risk of chronic and age-related degenerative diseases. It is cultivated in more than hundred countries and ranks as the world's seventh major food crop (*1*).

The vast amount of agricultural and food wastes has become a major concern throughout the world. Due to their composition with various beneficial ingredients, these wastes can be used in other production process as sources of dietary fibre, aromas, natural colours and antioxidants. However, sweet potato peels are considered major wastes generated during the processing of sweet potato with currently little commercial value. Sweet potato peels contain various classes of polyphenols and carotenoids with different health-promoting properties, which makes them suitable for processing into value-added ingredients in functional foods (*2*).

Generally, the recycling of agro-waste into high-value products could be achieved through solvent extraction technique. Phytochemicals in this form, especially carotenoids, are prone to destruction and oxidation from environmental factors such as oxygen, temperature and light. Therefore, some kind of processing or delivering system is required to ensure their effectiveness and target functions. Encapsulation is an effective method that improves the phytochemical stability by entrapping the core material with the coating agent (*3*). Among the different techniques, spray drying and freeze-drying are widely used in the food industry. Spray drying is commonly used due to its rapidity, flexibility and low cost, and freeze drying is especially effective for bioactive compounds susceptible to degradation at high temperatures. Compared with spray drying, freeze-drying has some disadvantages, including significantly higher processing times and higher unit cost (*4*).

Selection of appropriate coating material is very important, since it may influence encapsulation efficiency and stability of the encapsulated material. The most common wall materials are gum Arabic, maltodextrin, starch, sodium caseinate, gelatin, and their usability has been widely described in literature (*5*, *6*). However, many studies have indicated that whey protein isolate was very effective in encapsulating polar and non-polar compounds (*7*, *8*). Whey protein has a very high percentage of proteins (>90%), consisting mainly of β-lactoglobulin and α-lactalbumin, and it helps to reduce fat and maintain muscle mass in the human body (*9*). Also, whey protein exhibits good encapsulation ability due to its efficient emulsification, film- and gel-forming properties.

The final step of successful encapsulation is the incorporation of the obtained capsules in foods as a food additive, natural food colourant or nutrient supplement. Also, their storage stability requires testing (*10*). Spray and freeze-drying are processes with different temperature regimes and can lead to different physicochemical characteristics of encapsulated extract. Many factors such as light, temperature and oxygen are considered to influence their quality. Additionally, physicochemical characteristics such as moisture content, bulk density, particle size or encapsulation efficiency also impact the functional properties of capsules during storage.

The objective of this study is to investigate the possible utilization of sweet potato peel as a source of bioactive compounds, as well as the influence of spray and freeze-drying on the physicochemical properties of the obtained capsules using whey protein isolate as a carrier agent. Stability of the encapsulated bioactive compounds was evaluated during a 60-day storage period at ambient temperature ((25±5) °C) under light and dark conditions. The degradation kinetic analysis of encapsulated sweet potato peel extract and non-encapsulated extract was carried out during the mentioned period, where better retention of bioactive compounds was expected in the encapsulated extract.

## MATERIALS AND METHODS

### Plant material

Sweet potato was produced by Delta Agrar doo, Belgrade, Serbia, and purchased from the local store. The tubers were washed and manually peeled to a depth of approx. 1 mm. The flesh tubers and peel were blended separately in a kitchen blender (model Neo SK-400; TCL King Electrical Appliances Co., Ltd., Huizhou, PR China), freeze dried and stored at −20 °C until analysis.

### Chemicals and equipment

Chemicals used in the study were of analytical grade, purchased from Sigma Chemicals Co., Merck (St. Louis, MO, USA), J.T.Baker (Deventer, The Netherlands) and Lachner (Brno, Czech Republic). Distilled water was produced using water purification system DESA 0081 Water Still (POBEL, Madrid, Spain). The whey protein isolate was purchased from Olimp Laboratories (Debica, Poland). Absorbances in spectrophotometrical assays were measured on a Multiskan GO microplate reader (Thermo Fisher Scientific Inc., Waltham, MA, USA). For HPLC analysis, a Shimadzu Prominence chromatographic system (Kyoto, Japan) was used, which consisted of LC-20AT binary pump, CTO-20A thermostat and SIL-20A autosampler connected to the SPD-20AV UV/Vis detector. Freeze dryer, model Christ Alpha 2-4 LSC, was from Martin Christ, Osterode am Harz, Germany. Spray dryer, model Büchi mini B-290 was from Büchi Labortechnik, Flawil, Switzerland. High performance homogenizer (model Silent Crusher M) and shaker (model Unimax 1010) were from Heidolph Instruments GmbH, Kelheim, Germany. Centrifuge, model EBA 21, was from Hettich Zentrifugen, Tuttlingen, Germany.

### Extraction procedure

Sweet potato tubers and peel extracts were obtained following the method described by Šeregelj *et al.* (*7*). Briefly, freeze-dried samples were extracted three times using acetone/ethanol mixture (36:64) in solid to solvent ratio 1:20 for 10 min, with the same volume of solvents. The extraction was performed using a laboratory shaker at 300 rpm, under light protection, at room temperature. The three obtained extracts were filtered (Whatman paper No. 1), combined and stored in dark bottles at -20 °C until further analysis.

### Characterization of the sweet potato extracts

Total carotenoid content, expressed in mg of β-carotene per 100 g dry sample, was analyzed spectrophotometrically by the method of Nagata and Yamashita (*11*), using extraction solvent as the blank. The content of carotenoids was calculated using the following equation:

*w*(carotenoids as β-carotene)=0.216·*A*_663 nm_-1.22·*A*_645 nm_-0.304·*A*_505 nm_+0.452·*A*_453 nm_ /1/

where *A* is the absorbance measured at 663, 645, 505 and 453 nm.

Total phenolic content, expressed as gallic acid equivalents (GAE) per 100 g of sample dry mass, was determined spectrophotometrically by Folin-Ciocalteu method adapted to microscale (*12*). Briefly, the reaction mixture was prepared in 96-well microplate by mixing 15 μL extract, 170 μL distilled water, 12 μL the Folin-Ciocalteu reagent (2 M) and 30 μL of 20% sodium carbonate. After 1 h, the absorbance at 750 nm was measured using distilled water as a blank.

For HPLC analysis of carotenoids, solvent gradient was performed by varying the proportion of solvent A (water/methanol 1:4) to solvent B (acetone/methanol 1:1) at flow rates of 1 mL/min with the following gradient profile: 25% B 0-3 min, 75% B 3-6 min, 90% B 6-10 min, 100% B 10-18 min, 50% B 18-25 min, and 25% B 25-32 min. The column temperature was 25 °C and chromatograms were plotted at 450 and 475 nm. Carotenoids were dissolved in hexane and identified by comparing the retention time and its spectral characteristics with those of standards. Phenolic compounds were recorded using different wavelengths: 280 nm for hydroxybenzoic acids, 320 nm for hydroxycinnamic acids and 360 nm for flavonoids (*12*).

For all antioxidant assays, the results were expressed in μmol Trolox equivalent (TE) per 100 g sample dry mass. Three different methods were utilized: DPPH radical scavenging assay (*13*), reducing power (*14*) and β-carotene bleaching assay (*15*).

The DPPH radical scavenging assay was performed spectrophotometrically according to Gironés-Vilaplana *et al.* (*13*). Briefly, 250 μL DPPH^•^ solution in methanol (0.89 mM) were mixed with 10 μL of sample in a microplate well and left in the dark at ambient temperature. Absorbance was measured at 515 nm after 50 min. Methanol was used as a blank. DPPH radical scavenging activity values were calculated using the following equation:

DPPH radical scavenging activity=[(*A*_control_–*A*_sample_)/*A*_control_]·100 /2/

where *A*_control_ is the absorbance of the blank and *A*_sample_ is the absorbance of the extracts.

Reducing power was determined by the method of Oyaizu (*14*) adapted for 96-well microplate. In brief, 25 μL sample or 25 μL water (blank test), 25 μL sodium phosphate buffer, pH=6.6, and 25 μL of 1% potassium iron(III) cyanide were mixed and incubated in a water bath for 20 min at 50 °C. After cooling, 25 μL of 10% trichloroacetic acid were added and solutions were centrifuged at 2470×*g* for 10 min. After centrifugation, 50 μL supernatant were mixed with 50 μL distilled water and 10 μL of 0.1% iron(III) chloride in the microplate. Absorbances were measured immediately at 700 nm.

The β-carotene bleaching capacity of extracts was evaluated by the β-carotene linoleate model system of Al-Shaikan *et al*. (*15*). The absorbance of all the samples was taken at zero time at 470 nm and microplate was incubated at 50 °C during 180 min. Degradation rate (DR) was calculated using the following equation:

DR=ln(*A*_0 min_/*A*_180 min_)·1/*t* /3/

where *A*_0 min_ and *A*_180 min_ are the absorbances of sample at zero time and after 180 min, respectively, and *t* is the time when the reading was performed. Antioxidant capacity was calculated using the formula:

β-carotene bleaching capacity=[(DR_control_–DR_sample_)/DR_control_] ·100 /4/

where DR_control_ and DR_sample_ are the degradation rate of control and samsple, respectively.

### Encapsulation process

Freeze- and spray-dried capsules were prepared following the method described by Šeregelj *et al.* (*7*). A mass of 7 g of whey protein isolate was dissolved in 10 mL water at 60 °C and kept under stirring until the temperature reached 30 °C, while the mixture for spray drying was dissolved in the same way in 40 mL water. Separately, 40 mL sweet potato peel extract were combined with sunflower oil (1.5 mL), concentrated under reduced pressure on a rotary evaporator set at 40 °C to remove the organic solvent, and immediately mixed with previously prepared carrier solution.

The homogenized mixture was spray dried using a laboratory scale spray drier (Büchi mini B-290, Büchi Labortechnik) at an inlet temperature of 130 °C and an outlet temperature of (65±2) °C. The spraying air flow rate and rate of liquid feed were 600 L/h and 8 mL/min, respectively. Spray-dried capsules were packed in zip-lock plastic bags and stored at -20 °C for later analyses. For the freeze-drying method, the previously prepared mixture was kept overnight at -20 °C and then freeze dried (Christ Alpha 2-4 LSC; Martin Christ) at -40 °C for 48 h to ensure complete drying. Collected capsules were stored at -20 °C until further use.

### Characterization of the encapsulated sweet potato extracts

Water activity was determined by a LabSwift-aw meter (Novasina, Lachen, Switzerland) at 25 °C. The moisture content of the encapsulated extracts was measured using air oven method at 105 °C until a constant mass was obtained. For hygroscopicity, about 2 g of each capsule from the Petri dishes were placed at 25 °C in an airtight plastic container filled with NaCl saturated solution (75.29% RH). After one week, hygroscopic moisture (hygroscopicity) was weighed and expressed in g moisture per 100 g dry solids. Bulk (*ρ*_b_) and tapped density (*ρ*_t_) of the samples were determined using the method described in the European Pharmacopeia (*16*), and expressed in g/mL. For determination of *ρ*_b_, the sample (10 g) was poured into a measuring cylinder and the initial volume was noted as the bulk volume. The *ρ*_b_ was calculated according to the formula:

*ρ*_b_=*m*/*V*_b_ /5/

where *m* is the mass of the sample and *V*_b_ is the bulk volume of the powder.

For *ρ*_t_, the sample was tapped 250 times and then the volume was measured. Tapping was continued until the difference between successive volumes was less than 2% and this value was used as the tapped volume (*V*_t_)and *ρ*_t_ was calculated by the formula:

*ρ*_t_= *m*/*V*_t_ /6/

Carr’s Index and Hausner ratio were calculated according to the equations:

Carr’s index=[(*ρ*_t_–*ρ*_b_)/*ρ*_t_]·100 /7/

and

Hausner ratio=*ρ*_t_/*ρ*_b_ /8/

The particle size distribution of the obtained powders was determined using the Mastersizer 2000 laser diffraction size analyzer (Malvern Instruments, Worcestershire, UK) equipped with the Scirocco2000 dispersion unit. The size distribution was quantified as relative volume of particles in size bands presented as size distribution curves using Mastersizer 2000 software (*17*). Sample micrography was examined with HITACHI TM 3030 scanning electron microscope (SEM). The colour measurements were made with a Minolta reflectance colourimeter (Minolta ChromaMeter CR-300; Minolta, Osaka, Japan) considering the CIELab colour system. Chroma or saturation (C*) was calculated according to the following formula:

C*=√(a*^2^+b*^2^) /9/

Mass fractions of surface carotenoids and total carotenoids in the encapsulated sweet potato peel extracts, as well as the encapsulation efficiency (EE) of carotenoids were determined according to the method of Barbosa *et al.* (*18*). Surface carotenoid content was determined by direct extraction of 0.25 g encapsulated sample with 5 mL acetone on a vortex for 20 s, followed by centrifugation at 6805×*g* (10 min) and supernatant separation. For total carotenoid content determination, 0.25 g sample was vortexed with 0.2 M phosphate buffered saline (PBS, pH=7) for 1 min to break the capsules, extracted with 2 mL acetone and 3 mL diethyl ether. After separating the pigment layer, extraction was repeated with the same solvent volumes to collect total pigment. The carotenoid quantification was carried out according to the previously described procedure. The EE of carotenoids was calculated by using the equation:

EE(carotenoids)=[(*w*(total carotenoids)-*w*(surface carotenoids))/*w*(total carotenoids)]·100 /10/

Simultaneously, the control sample, *i.e.* the carrier (whey protein) without extracts, was prepared in the same way for the correction of interfering substances originating from the carrier material.

Mass fractions of surface phenolics and total phenolics, and EE of phenolics in the encapsulated sweet potato peel extractswere determined according the methods described by Tumbas Šaponjac *et al.* (*12*). For the total phenolics, 100 mg sample were dispersed in 1 mL ethanol, acetic acid and water (50:8:42). The mixture was then vortexed for 1 min, centrifuged at 6805×*g* for 2 min, and the supernatant was separated. Similarly, for surface phenolics, 100 mg sample were dispersed in 1 mL ethanol and methanol (1:1) mixture. The mixture was vortexed for 1 min, centrifuged at 6805×*g* for 2 min, and the supernatant was separated. The surface and total phenolics were determined by Folin–Ciocalteu method described above. The EE of phenolics was determined by using the equation:

EE(phenolics)= [(w(total phenolics)-*w*(surface phenolics))/*w*(total phenolics)]·100 /11/

The corrections for interfering substances originating from whey protein were made by simultaneously preparing, in the same manner, control samples in which the encapsulated extract was replaced with the matching concentration of whey protein.

### Storage stability of encapsulated bioactive compounds

The sweet potato peel extract, spray-dried and freeze-dried encapsulated extracts were stored at ambient temperature (25±5 °C) in glass and amber bottles for 60 days to determine the effects of time and light exposure on the stability of bioactive compounds. For that purpose, total carotenoids and total phenolics in the extract and encapsulated extract were determined by previously described methods every 15 days. Bioactive compound retention was calculated by the formula:

Carotenoid or phenolic retention=[*w*(total carotenoids or phenolics at *t*(storage))/*w*(total carotenoids or phenolics at *t*_0_(storage)]·100 /12/

Rate constant (*k*) and half-life time (*t*_1⁄2_) were calculated by the method of Cai and Corke (*19*) using the regression analysis of ln (pigment retention) against storage time when plotted on a natural logarithmic scale (*20*).

### Statistical analysis

All experiments were run in triplicate. The results are presented as mean value±standard deviation (S.D), *N*=3. Statistical analyses were carried out using Origin v. 8.0 SRO software package (*21*) and demo version of the MS Office XLSTAT-Pro 2014 (*20*) statistical package. Significant differences were calculated by ANOVA (p<0.05).

## RESULTS AND DISCUSSION

### Chemical characterization of the sweet potato extracts

Efficient exploitation of agro-waste as a rich and inexpensive source of beneficial phytochemicals in different commercial sectors such as food, pharmaceutical, and cosmetic industries requires an appropriate method of extraction of these bioactive compounds from plant materials. Sweet potato contains various types of phytochemicals with different polarities, and there is no single solvent optimal for their extraction. The effect of solvents (acetone and acetone/ethanol mixture) on the extraction efficiency of total carotenoids and phenolics from sweet potato flesh tuber and peel, and the antioxidant activity of the extracts were investigated. The mass fractions of total carotenoids and total phenolics of sweet potato extracts are given in Table 1.

**Table 1:** The mass fractions of bioactive compounds and antioxidant capacity on dry mass basis of sweet potato tuber and peel extracts

**Sweet potato extract**	***w*(total carotenoids as β-carotene)/(mg/100 g)**	***w*(total phenolics as GAE)/(mg/100 g)**	**DPPH radical scavenging activity**	**Reducing power**	**β-Carotene bleaching capacity**
*n*(TE)/(*m*(sample))/(μmol/100 g)		
**Tuber** **(Acetone)**	(19.5±0.3)^c^	(50.7±2.0)^a^	(239.0±4.0)^a^	(48.8±1.6)^a^	(7353.7±12.4)^b^
**Tuber (Acetone+ethanol)**	(19.67±0.08)^c^	(179.8±0.9)^c^	(500.0±2.9)^c^	(436.2±2.1)^c^	(6567.7±72.5)^a^
**Peel** **(Acetone)**	(12.0±0.3)^a^	(130.9±1.1)^b^	(431.3±9.8)^b^	(80.6±1.0)^b^	(12987.8±47.7)^d^
**Peel (Acetone+ethanol)**	(12.71±0.05)^b^	(262.3±1.8)^d^	(615.2±2.3)^d^	(506.7±3.9)^d^	(12317.9±15.7)^c^

Data represent mean value of three replicates±S.D. Mean values with different letters in superscript in columns are significantly different (p<0.05). GAE=gallic acid equivalent, TE=Trolox equivalent

Carotenoid (as β-carotene) mass fraction on dry mass basis in the analysed sweet potato extracts ranged from 12.00 to 19.67 mg/100 g without significantly different yield (p<0.05) among different extraction solvents used for the same tuber parts. Expectedly, the sweet potato tuber extracts had higher β-carotene content (p<0.05) than peel extracts, indicating that the deep orange colour of the tuber flesh is positively correlated with the β-carotene content. Kammona *et al.* (*22*) tested various varieties of sweet potato tuber flesh of orange, yellow, purple and white colours. They reported that four varieties of different colour flesh had highly significant differences in total and individual carotenoid content. The orange sweet potato had the highest (389.22 µg/g) total carotenoid content, followed by yellow (138.96 µg/g), purple (116.28 µg/g) and white (115.18 µg/g) potato. Rose and Vasanthakaalam (*23*) reported 13.1 mg/100 g β-carotene in fresh mass of orange varieties. Yellow varieties 'Kwizekumwe' and '440170' yielded β-carotene on dry mass sample 1.85 and 1.68 mg/100 g, whereas white potato did not contain β-carotene. It is reported that the flesh colour and β-carotene mass fraction in sweet potato vary widely because they are affected by variety, maturity, post-harvest storage conditions, season and the part of the tuber consumed (*24*).

Mass fraction of total phenolics in sweet potato tuber flesh and peel extracts varied significantly (p<0.05) between the two extraction solvents tested. Total phenolic yields expressed as GAE of tuber and peel acetone extracts were 50.7 and 130.9 mg/100 g, which is more than 2-3 times lower than the yield of acetone/ethanol extracts (Table 1). Silla *et al.* (*25*) reported that ethanol has a hydroxyl group, which forms intramolecular hydrogen bond with the hydroxyl group present in phenolic compounds and increase their solubility. Higher mass fraction of phenolics in sweet potato peel than in the tuber extracts were also reported by other researchers (*26*). This observation can be related to the fact that phenolic compounds are mostly concentrated in the epidermal and sub-epidermal layers of the plants due to their protective role against UV radiation as well as pathogen and pest attack (*27*).

Free radicals produced in the body play part in several health disorders in humans, including atherosclerosis, arthritis, ischemia, central nervous system injury, gastritis, and cancer. Due to their scavenging activity, antioxidant phytochemicals are able to reduce the risk of many chronic diseases. The stable DPPH free radical has been widely used to test the free radical scavenging activity of various antioxidant phytochemicals. In addition, reducing power of bioactive phytochemicals was associated with antioxidant activity, since it is related to their ability to transfer electrons. Acetone/ethanol extracts of sweet potato peel and tuber exhibited higher (p<0.05) free radical scavenging activity in DPPH^•^ test, as well as reducing power (Table 1). The increasing trend of antioxidant activity followed the increase of the mass fraction of total phenolics. According to Khlifi *et al.* (*28*), the presence of phenolics was associated with a wide range of biological activities including antioxidant properties. β-Carotene bleaching assay measures the loss of the yellow colour of β-carotene due to its reaction with formed linoleic acid radicals caused by oxidation. All sweet potato extracts prevented the bleaching of β-carotene in β-carotene-linoleic acid mixtures with varying degrees of antioxidant capacity. Acetone and acetone/ethanol extracts of sweet potato peel were more effective in the inhibition of lipid peroxidation (approx. twice higher than the tuber extracts). Different composition of antioxidant compounds in the extracts exhibited a different rate of β-carotene bleaching. Thus, extracts with higher content of non-polar antioxidants showed lower antioxidant capacity during β-carotene bleaching. Similar trend was observed by Oke Altunas *et al.* (*29*) and Dorta *et al.* (*30*).

Characterization of individual phenolics and β-carotene in sweet potato peel extracts was conducted by HPLC method (Table 2). β-Carotene is the predominant carotenoid in orange varieties of sweet potato (*22*). The highest amount of β-carotene quantified by HPLC analysis was in the acetone/ethanol extract of sweet potato tuber (17.9 mg/100 g), but not significantly different (p<0.05) from the acetone extract (16.3 mg/100 g). β-Carotene in Indonesian and Malaysian orange sweet potatoes were detected in higher mass fractions (405.07 and 938.08 μg/g) (*22*). Rodriguez-Amaya *et al.* (*31*) reported that tropical climate elevates carotenoid biosynthesis, which explains the differences in β-carotene contents.

**Table 2:** The mass fraction of β-carotene and individual phenolic compounds on dry mass basis in sweet potato tuber and peel extracts

**Compound**	**Tuber** **(acetone)**	**Tuber** **(acetoneethanol)**	**Peel** **(acetone)**	**Peel** **(acetoneethanol)**
*w*/(mg/100 g)			
**β-carotene**	(16.3±1.4)^a^	(17.9±0.7)^a^	(10.4±0.2)^b,c^	(11.5±0.7)^c^
**Gallic acid**	(5.7±0.3)^a^	(22.0±0.9)^b,c^	(20.9±1.0)^b^	(23.9±0.6)^c^
**Catechin**	(18.9±0.2)^a^	(98.1±3.7)^c^	(65.7±0.9)^b^	(114.6±1.3)^d^
**Epicatechin**	(3.77±0.04)^a^	-	(9.0±0.5)^b^	-
**Vanillic acid**	(4.8±0.1)^a^	(27.8±0.6)^c^	(14.41±0.02)^b^	(34.1±0.3)^d^
**Caffeic acid**	(5.33±0.05)^b^	(22.9±0.2)^d^	(2.29±0.02)^a^	(20.46±0.05)^c^
**Coumaric acid**	(1.66±0.01)^a^	(3.25±0.07)^b^	-	(3.56±0.01)^c^
**Rutin**	(1.76±0.01)^b^	(1.50±0.01)^a^	(14.1±0.1)^d^	(10.18±0.09)^c^

Data represent mean value of three replicates±S.D. Mean values with different letters in superscript in rows are significantly different (p<0.05)

Several phenolic compounds were identified (Table 2), *i.e*. two hydroxybenzoic acids (gallic and vanillic), two hydroxycinnamic acids (caffeic and coumaric), two flavan-3-ols (catechin and epicatechin), and one flavonol (rutin). Catechin is the most abundant phenolic compound in all sweet potato extracts, and higher mass fraction was found in acetone/ethanol tuber and peel extracts (98.1 and 114.6 mg/100 g, respectively). On the other hand, epicatechin was found in acetone and extracts, while it was not detected in acetone/ethanol extracts. Ayeleso *et al.* (*32*) also reported the importance of the influence of extraction solvents on the level of phenolics in plants, as well as the presence of catechin, caffeic acid, vanillic acid and rutin in sweet potato.

### Physicochemical characterization of the encapsulated sweet potato extracts

Since the acetone/ethanol extract of sweet potato peel stood out in terms of antioxidant properties, it was chosen for encapsulation evaluation by two different techniques (spray and freeze-drying). The spray and freeze-drying of the extract with whey protein isolate as the wall material caused significant differences in the properties of the encapsulated extract (Table 3). ANOVA showed significant differences (p<0.05) in all the assessed traits among the samples.

**Table 3:** Physical properties of encapsulated sweet potato peel extracts

**Characteristic**	**Spray-dried extract**	**Freeze-dried extract**
***a*_w_**	(0.089±0.00)^b^	(0.021±0.00)^a^
***w*(moisture)/%**	(3.73±0.00)^b^	(1.17±0.00)^a^
**((*m*(water)/*m*(encapsulate))/(g/100 g)**	(7.96±0.01)^b^	(6.89±0.00)^a^
***ρ*_b_/(g/mL)**	(0.45±0.00)^b^	(0.34±0.03)^a^
***ρ*_t_/(g/mL)**	(0.77±0.02)^b^	(0.52±0.02)^a^
**Carr’s index/%**	(40.91±0.05)^b^	(34.5±1.0)^a^
**Hausner ratio**	(1.69±0.01)^b^	(1.53±0.01)^a^
***D*_m_/μm** ***d*(0.1)** ***d*(0.5)** ***d*(0.9)**	36.99^a^ 4.89^a^ 12.53^a^ 101.60^a^	203.60^b^ 39.52^b^ 176.71^b^ 400.99^b^
**CIE Lab** L* a* b* C*	(91.42±0.01)^b^ (-1.84±0.01)^a^ (17.75±0.02)^a^ (5.64±0.01)^a^	(82.25±0.03)^a^ (0.12±0.02)^b^ (37.06±0.02)^b^ (8.62±0.02)^b^
**EE(carotenoids)/%**	(60.0±0.2)^b^	(9.34±0.03)^a^
**EE(phenolics)/%**	(61.9±0.4)^a^	(64.3±0.2)^b^

*ρ*_b_ and *ρ*_t_ are bulk and tap densities, respectively, *D*_m_ is volumetric mean diameter, and EE is encapsulation efficiency. Data represent mean value of three replicates±S.D. Mean values with different letters in superscript in rows are significantly different (p<0.05)

In the fields of food science and safety, water activity and moisture content are important parameters due to their role in lipid peroxidation, microbial growth, and enzymatic and non-enzymatic reactions in food. High water activity indicates more free water available for biochemical reactions, which affects the shelf life of food. Generally, there is no microbial growth in products with *a*_w_ below 0.6, which are classified as dehydrated food. The obtained *a*_w_ values of encapsulated sweet potato peel extracts are indicators of microbiologically stable environments (*a*_w_(spray-dried extract)=0.089 and *a*_w_(freeze-dried extract)=0.021) (Table 3). Higher water activity of spray-dried than of freeze-dried extract is observable, which is comparable with the results published by Kuck and Noreña (*33*).

Moisture content represents the water composition of a system, which is related to the drying efficiency. The lower moisture level, which means better preservation and stability of the encapsulated product, was noticed after freeze-drying (1.17%) (Table 3). Percentage of moisture after spray drying was also in the range of microbiological stability (3.73%). Generally, higher moisture levels of encapsulated protein than of other wall materials might be attributed to very hygroscopic nature of whey protein isolate.

According to Jaya and Das (*34*), the hygroscopicity is defined as the ability of food powder to absorb moisture from the environment. Hygroscopic behaviour of the encapsulated material is very important for food technology, because it is necessary to find the best techniques to obtain a product with stable properties to withstand the storage conditions. The higroscopicity value is related to the moisture content (*35*), where higher moisture content means a higher higroscopicity value. After seven days, higher hygroscopicity was reached in spray-dried extract (7.96 g of water per 100 g of encapsulated material) than in freeze-dried extract (6.89 g of water per 100 g of encapsulated material) (Table 3). The obtained results confirm the statement made by Nawi *et al.* (*35*).

Density and compressibility play a significant role in food packaging, since they affect the flowability and storage stability of the encapsulated material. The higher values of bulk and tapped density were observed for spray-dried extract (0.45 and 0.77 g/mL) (Table 3), which may be attributed to their smaller particle size. Kshirsagar *et al.* (*36*) also reported that bulk density of spray-dried encapsulated extract was affected by the particle size and sphericity of granules. The high bulk and tapped densities of encapsulated extract enable the use of smaller containers for packing and reduce the possibility of powder oxidation.

Flow properties of the encapsulated material are generally assessed by Carr’s index and Hausner ratio. The Carr’s index or compressibility index is defined as the indication of the compressibility of encapsulated material, and the Hausner ratio is correlated with its flowability. The obtained values of Carr index and Hausner ratio were greater than 34 and 1.45 (Table 3), respectively, which indicates poor flowability of both samples. Generally, the flow property depends on the wall material properties applied for encapsulation. Thus, in the study of Kulthe *et al.* (*37*), β-carotene encapsulated with maltodextrin as wall material showed good flow characteristics, while β-carotene encapsulated with potato starch and gelatin showed fair flow characteristics. According to Zhang *et al.* (*38*), encapsulated material with good flow properties is convenient for handling and processing operations.

Table 3 shows the particle size distribution of encapsulated sweet potato peel extracts. Smaller diameter, from 4.89 to 101.60 μm, of spray-dried extracts is observable. The freeze-dried extract had significantly greater particle size (39.52-400.99 μm), which can be explained with low process temperature, and the lack of strength to break the frozen drops or to alter the surface during drying (*39*). The study of Kuck and Noreña (*33*) about encapsulation of grape skin extract by spray and freeze-drying also showed greater particle size formed during freeze-drying.

Colour properties of encapsulated sweet potato peel extracts were determined on the basis of L*, a* and b* parameters. The high L* values (close to 100%) indicate a very light colour of both encapsulated samples. Spray-dried extract was characterized by the lowest L* (Table 3), which may be explained by smaller particle size and higher temperature during encapsulation. The high temperature of spray drying leads to the destruction of surface carotenoids, and a decrease in the reddness and yellowness (a* and b* values). On the other hand, formation of ice crystals during freezing alters the pore structure after water sublimation. Thus, the surface of these particles is darker due to light scattering properties of the empty spaces after sublimation (*33*). The chroma (C*), or degree of colour saturation, of the freeze-dried extract was higher, which confirmed that this sample has higher colour intensity. Similar results were found for the encapsulated mango pulp (*40*) and pumpkin seed oil (*41*).

In general, encapsulation efficiency reflects the capability of wall material to encapsulate the target compounds. Spray drying provided the higher EE of carotenoids (60.0%) than freeze-drying (9.34%) (Table 3). Considering that the freeze-drying technique provides the encapsulated products with a larger particle diameter, there are signifficant amounts of unencapsulated carotenoids at the surfaces of big particles. Surface carotenoids are more susceptible to alterations caused by the external environment than the encapsulated or total carotenoids. On the other hand, encapsulation efficiency of phenolics was 61.9 and 64.3% by spray and freeze-drying, respectively. Differences among the results may be explained by the fact that encapsulation efficiency strongly depends on the encapsulated compounds, wall material used, as well as encapsulation technique (*42*).

**Fig. 1:**
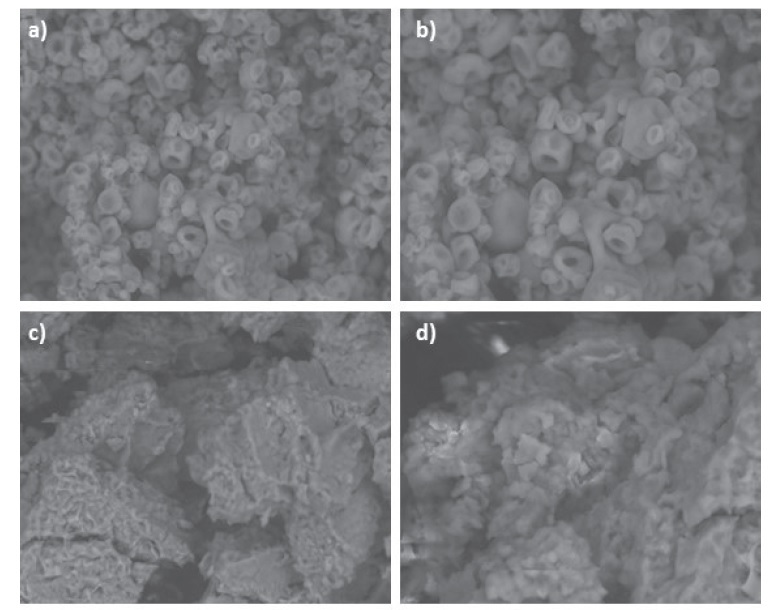
Scanning electron micrographic properties at magnification of: a) 1000× and b) 2000× of spray-dried encapsulated extracts, and c) 1000× and d) 2000× of freeze-dried encapsulated extracts

The micrographic analysis of encapsulated sweet potato peel extractswas done by scanning electron microscopy (SEM) and the results are in Fig. 1. SEM results of the encapsulated extracts prepared by spray drying show separated, non-spherical particles, with varying diameters and concavities (Figs. 1a and 1b). The formation of concavities on the surfaces of the microparticles was attributed to the shrinkage of the particles due to the dramatic loss of moisture after cooling (*43*, *44*). These results are in accordance with Šeregelj *et al*. (*45*) for encapsulation of red pepper waste bioactive compounds performed by the same technique and wall material. By contrast, the freeze-dried encapsulates extracts showed a completely different morphology of particles mostly larger in diameter, with irregular shapes, pores and wrinkles (Figs. 1c and 1d). This could be explained by the low temperature involved in the freeze-drying process, which results in the lack of forces for breaking up the frozen liquid into droplets (*39*).

The mass fractions of β-carotene and individual phenolic compounds in encapsulated sweet potato peel extracts are shown in Table 4. Similarly to the phenolics in the acetone/ethanol extract of sweet potato peel, catechin has the largest mass fraction in both encapsulated samples. Higher bonding affinity of whey protein to phenolic compounds than of β-carotene is observable. The significantly higher (p<0.05) mass fraction of β-carotene was found in the freeze-dried extract, with larger amount bound on the surface of particles as previously determined in this study.

**Table 4:** The mass fractions of β-carotene and individual phenolic compounds on dry mass basis in the encapsulated sweet potato peel extracts

**Compound**	**Spray-dried extract**	**Freeze-dried extract**
*w*/(mg/100 g)	
**β-carotene**	(1.93±0.01)^a^	(2.06±0.02)^b^
**Gallic acid**	(2.67±0.01)^b^	(2.41±0.03)^a^
**Catechin**	(5.68±0.01)^b^	(5.40±0.06)^a^
**Epicatechin**	-	-
**Vanillic acid**	(2.18±0.00)^b^	(1.84±0.01)^a^
**Caffeic acid**	(1.73±0.02)^b^	(1.62±0.01)^a^
**Coumaric acid**	(0.11±0.00)^b^	(0.06±0.00)^a^
**Rutin**	(0.09±0.00)^a^	(0.09±0.00)^a^

Data represent mean value of three replicates±S.D. Mean values with different letters in superscript in rows are significantly different (p<0.05)

### Storage stability studies

**Fig. 2:**
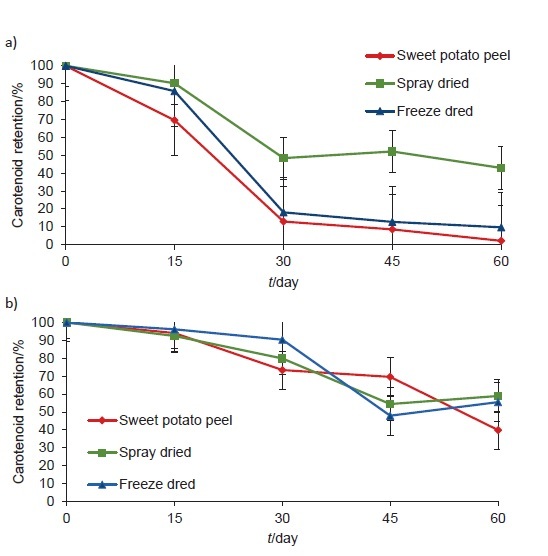
Carotenoid retention of sweet potato peel extracts, spray-dried and freeze-dried encapsulated sweet potato peel extracts during a 60-day storage at ambient temperature ((25±5) °C) under: a) light and b) dark conditions

The carotenoid retention of spray- and freeze-dried sweet potato peel extracts was monitored during 60 days of storage under ambient (25±5) °C light and dark conditions (Fig. 2). Generally, under the light conditions, all samples retained smaller amount of carotenoids. Carotenoids are prone to degradation, more precisely to isomerisation, especially at high temperature, in daylight, and oxidation due to the occurrence of oxygen in food. Under those conditions, *trans* isomer of carotene is converted into *cis* isomer, which is much more susceptible to oxidation (*31*).

The highest average loss of the carotenoids occured between the 15th and 30th day of storage. Under both light and dark storage conditions, spray-dried extracts showed the best carotenoid retention, *i.e.* 42.91 and 58.88%, respectively (Fig. 2). On the other hand, freeze-dried extracts showed similar retention under dark conditions (55.5%), while a significant decrease in carotenoid content and low retention occurred under light storage (9.73%). This observation could be explained with higher surface carotenoid content of the freeze-dried extracts, which leads to its more rapid degradation. As expected, the lowest carotenoid retention was observed in the sweet potato peel extract, and reached 2.18 and 39.86% under light and dark conditions respectively.

The carotenoid degradation kinetics followed first-order kinetics, which is indicated by linear regression of ln (carotenoid retention) with negative slope when plotted on a natural logarithmic scale (Table 5). Linear correlation coefficients (R^2^) ranged from 0.75 to 0.90. Rate constants for light and dark storage conditions of spray-dried extract were 0.0149 and 0.0106 day^-1^, respectively, and half-life values were 46.52 and 65.39 days. The lowest half-life values were observed for sweet potato peel extracts (11.14 and 49.50 days, respectively), which indicates that encapsulation increased pigment retention during storage. Similar results were observed for commercial β-carotene encapsulated in maltodextrin (*46*). The degradation kinetics of carotenoids in pumpkin slices has been studied as well (*47*).

**Table 5:** Kinetic parameters of carotenoid degradation in sweet potato peel extract and encapsulated extract during 60 days of storage under ambient light and dark conditions

**Sample**	**y**	**R^2^**	***k*/day^-1^**	***t*_1/2_/day**
**Light condition**				
**SPP extract**	4.7338-0.0622x	0.88	0.0622	11.14
**SDE**	4.5882-0.0149x	0.84	0.0149	46.52
**FDE**	4.6679-0.0438x	0.90	0.0438	15.83
**Dark condition**				
**SPP extract**	4.7519-0.0141x	0.76	0.0014	49.50
**SDE**	4.6349-0.0106x	0.87	0.0106	65.39
**FDE**	4.687-0.0125x	0.75	0.0125	55.45

SPP=sweet potato pee, SDE=spray dried encapsulates, FDE=freeze dried encapsulates, R^2^= linear correlation coefficient, *k*=rate constant, *t*_1/2_=half-life time. Data present mean value of three replicates±S.D.

Phenolic compounds, in contrast to carotenoids, exhibited much higher retention in the same storage period in all investigated samples. Their degradation in spray-dried extracts stored under dark conditions was the lowest (5.24%), while it was the highest in the sweet potato peel extract under light conditions (18.59%) (data not shown). Our results are in accordance with Tumbas Šaponjac *et al.* (*12*), where phenolics in freeze-dried extract of beetroot pomace increased for 19.08% during two months. Castro-López *et al.* (*48*) demonstrated that phenolic compounds in different juice samples did not decrease until 20 days of storage. These findings are in agreement with the observation of Kevers *et al.* (*49*), who reported that the phenolic compounds of many fruits and vegetables remain stable during storage.

## CONCLUSIONS

Determination of mass fractions of total and individual phenolics and carotenoids in sweet potato tuber and peel extracts revealed that sweet potato peel extract had higher mass fraction of total phenolics and higher antioxidant activity than sweet potato tuber extract, indicating that removal of peel from sweet potato may induce significant nutrient loss. Due to the high rate of biodegradation, isolated bioactive compounds can be encapsulated to prolong their storage stability and beneficial properties. Freeze drying method provides the better quality of encapsulates in terms of water activity, moisture content, hygroscopicity, and encapsulation efficiency (EE) of phenolics. Spray drying provided encapsulated material with lower particle size, better flow properties, and EE of carotenoids, which are very important parameters for long-term stability. A high temperature of spray drying affected the colour properties of the encapsulated material, which had higher L* (lightness) than the freeze-dried sample. Spray drying enables the carotenoid and phenolic compound retention and prolonged shelf life under light and dark conditions. Overall, the obtained results showed that spray drying and use of sweet potato peel are efficient for functional food development, with improved nutritional, colour and bioactive properties.
